# World Stroke Organization mechanical thrombectomy workshops combined with the Global Stroke Alliance builds regional capacity

**DOI:** 10.3389/fneur.2026.1866027

**Published:** 2026-10-02

**Authors:** Violiza Inoa, Ryna Then, Nicole M. Cancelliere, Nitin Goyal, Karelle Spence, Michael J. Wells, Andres Fonnegra, Omar Lopez, Fernando Gonzalez, Daniel Mantilla, Alex Eusebio, Cedric Pimentel, Marc Ribo, Manuel Moreu, Serdar Geyik, Ossama Mansour, Songul Senadim Kaytanci, Cagatay Andiç, Atilla Ozcan Ozdemir, Vipul Gupta, Vikram Huded, Octavio Marques Pontes-Neto, Gaurav Goel, Erdem Gurkas, Semih Giray, Raul Nogueira, Laetitia de Villiers, Ruchi Kabra, Rajsrinivas Parthasarathy, Vitor Mendes Pereira, Vu Dang Luu, Jeyaraj Pandian, Sheila Martins, Gillian Gordon Perue

**Affiliations:** 1 Semmes-Murphey Clinic, University of Tennessee Health Science Center, Memphis, TN, United States; 2 Neurology Division, Jefferson Einstein Philadelphia Hospital, Philadelphia, PA, United States; 3 St Michaels’s Hospital, Toronto, ON, Canada; 4 Jackson North, Miami, FL, United States; 5 Miller School of Medicine, University of Miami, Miami, FL, United States; 6 Shaio Clinic Foundation, Bogota, Colombia; 7 Department of Neurosurgery, Johns Hopkins University School of Medicine, Baltimore, MD, United States; 8 Autonomous University of Bucaramanga, Bucaramanga, Colombia; 9 Northwell Health, New York, NY, United States; 10 Division of Neurocritical Care, Department of Neurology and Neurosurgery, Emory University School of Medicine, Atlanta, GA, United States; 11 Hospital Vall d’Hebron, Barcelona, Spain; 12 Neurointerventional Unit, Hospital Clinico Universitario San Carlos, Madrid, Spain; 13 Department of Radiology (S.G.), IAU Stroke Center, Istanbul Aydin University, Istanbul, Türkiye; 14 Department of Neurology, Alexandria University, Alexandria, Egypt; 15 Department of Neurology, Bakırköy Research and Training Hospital for Psychiatry, Neurology, Neurosurgery, Istanbul, Türkiye; 16 Department of Interventional Radiology, Ba¸skent University Dr. Turgut Noyan Application and Research Center, Adana, Türkiye; 17 Eskisehir Osmangazi University, Eskişehir, Türkiye; 18 Department of Neurointerventional Surgery, Sir H. N. Reliance Foundation Hospital and Research Centre, Mumbai, Maharashtra, India; 19 Department of Interventional Neurology, Mazumdar Shaw Medical Center, Narayana Health Institute of Neuroscience, Bengaluru, India; 20 Department of Neurosciences and Behavioral Sciences, Ribeirão Preto Medical School, University of São Paulo, Ribeirão Preto, Brazil; 21 Department of Neurointervention Surgery, Neurointervention Surgery, Institute of Neurosciences, Medanta, The Medicity Gurugram, Gurugram, Haryana, India; 22 Department of Neurology, Dr Lutfi Kirdar City Hospital, Istanbul, Türkiye; 23 Department of Neurology, Gaziantep University, Gaziantep, Türkiye; 24 Departments of Neurology, Neurosurgery, and Radiology, Marcus Stroke and Neuroscience Center, Grady Memorial Hospital, Emory University School of Medicine, Atlanta, GA, United States; 25 Department of Interventional Neuroradiology, Gold Coast University Hospital, Southport, QLD, Australia; 26 Neurological Intervention and Imaging Service of Western Australia (NIISWA), Perth, WA, Australia; 27 Paras Health, Gurugram, Haryana, India; 28 St Michaels’ Hospital, Toronto, ON, Canada; 29 Stroke Center Bach Mai Hospital, Hanoi, Vietnam; 30 Department of Neurology, Christian Medical College and Hospital Ludhiana, Ludhiana, India; 31 Universidade Federal do Rio Grande do Sul, Porto Alegre, Brazil

**Keywords:** hands on simulation, low to middle income countries, mechanical thrombectomy, neurointerventional physician education, training

## Abstract

**Introduction:**

Access to mechanical thrombectomy (MT) remains limited in many low- and middle-income or resource-limited regions because of gaps in trained personnel, procedural experience, public awareness, and stroke-system readiness. The World Stroke Organization paired simulation-based MT workshops with the Global Stroke Alliance to combine hands-on neurointerventional education with regional advocacy, Ministry of Health engagement, public awareness activities, and stakeholder collaboration. This study evaluated the feasibility, scalability, and self-reported educational impact of MT workshops delivered within this broader WSO/GSA capacity-building framework.

**Methods:**

A comprehensive 22-h curriculum was developed, incorporating didactic sessions, hands-on simulations, and complex case studies in collaboration with the Global Stroke Alliance between August 2023 and July 2025. The workshops were designed for neurointerventional physicians and tailored to regional needs. Pre- and post-course assessments measured physician confidence in MT procedures, while a three-month follow-up survey evaluated changes in MT volumes and clinical practice. Data were analyzed using summary statistics, chi-square tests, and McNemar’s tests as appropriate.

**Results:**

Among 112 participating physicians from 24 countries, 81% completed the pre-test and 72% completed the post-test; 67% had paired pre- and post-test responses. The proportion of participants rating themselves as “very comfortable” increased from 15% pre-workshop to 41% post-workshop (*p* < 0.001), with notable improvements in patient selection for MT (15–48%, *p* < 0.001), procedural access knowledge (15–46%, *p* < 0.001), thrombectomy technique (9–41%, *p* < 0.001), and avoiding harmful maneuvers (7–33%, *p* < 0.001). At 3 months, 32 participants completed follow-up, of whom 37.5% self-reported an increase in overall thrombectomy numbers.

**Conclusion:**

Simulation-based MT workshops are feasible and scalable across multiple regions and were associated with improved self-reported procedural confidence and early self-reported changes in clinical practice. These findings support structured hands-on training as one component of broader capacity-building efforts, while highlighting the need for objective procedural volume, patient outcome, and registry-based data to determine the effect of such programs on MT access and stroke care delivery.

## Graphical abstract

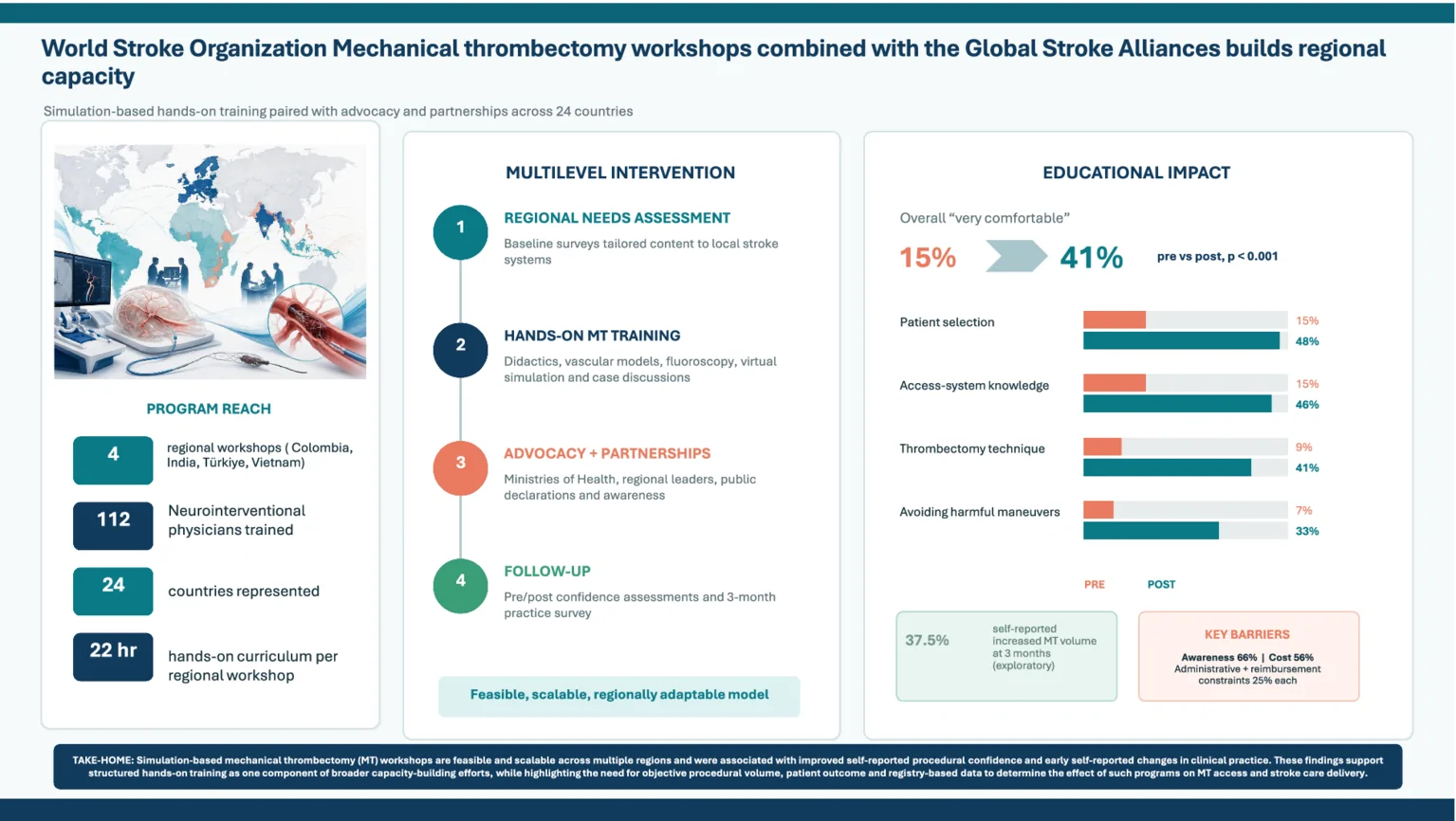

## Introduction

Mechanical thrombectomy (MT) is an established and highly effective intervention for acute large-vessel ischemic stroke (1). Despite its proven benefit, access to MT remains limited in many low- and middle-income or resource-limited regions because of gaps in trained personnel, procedural experience, public awareness, referral pathways, reimbursement, and stroke-system readiness (2). The World Stroke Organization (WSO) has prioritized reducing stroke-related death and disability, with a particular focus on LMICs. Over the past four decades, stroke incidence has declined by more than 42% in high-income countries but has increased by 100% in LMICs, underscoring a growing global disparity. Stroke also imposes a substantial socioeconomic burden in these countries (3). These trends highlight the need for targeted educational strategies that build MT capacity while also addressing system-level barriers through regional advocacy and stakeholder engagement.

To address this gap, the World Stroke Organization paired simulation-based MT workshops with the Global Stroke Alliance to combine hands-on neurointerventional education with Ministry of Health engagement, public awareness activities, regional advocacy, and stakeholder collaboration.

This study evaluates the feasibility, scalability, and self-reported educational impact of MT workshops delivered within this broader WSO/GSA capacity-building framework. These simulation trainings were paired with the WSO Global Stroke Alliance (GSA), which engages Ministries of Health, local stroke associations, and regional leaders to simultaneously strengthen networking, public awareness, and stroke care infrastructure in the region. The GSA framework typically includes a public declaration on the fight against stroke, signed by the host country’s health minister and often covered by local media. Health ministers from other invited countries in the region are also typically invited, further amplifying awareness and policy engagement across the region.

## Materials and methods

In this paper, we report on the design, implementation, and evaluation of mechanical thrombectomy (MT) skills workshops conducted in Colombia (August 2023) as well as additional workshops held in conjunction with the GSA in India (September 2023), Türkiye (October 2024), and Vietnam (July 2025). These workshops were delivered in collaboration with the World Stroke Organization (WSO) Global Stroke Alliance (GSA). The primary objective was to evaluate the feasibility, scalability, and self-reported educational impact of simulation-based MT workshops delivered within a broader WSO/GSA capacity-building framework.

### Curriculum development process

Building on the foundations of our previously published workshops (4), we used the survey data to design tailored curricula aimed at strengthening stroke infrastructure in each region. Prior to curriculum development and customization for each region, baseline surveys were distributed to prospective participants to assess the regional stroke treatment landscape and institutional capacity. Survey items evaluated the availability of stroke care services, current utilization of MT, and perceived barriers to implementation (see Supplementary material). These data served as the foundation for tailoring workshop content to address region-specific needs and gaps. Workshop regions were selected based on the WSO’s existing working relationship with the Ministry of Health in the host country. After the host country was identified, other countries within the same region were invited to participate, allowing the workshop to address regional MT capacity needs while leveraging established governmental and stroke-society partnerships.

The tailored training curriculum comprised a 22-h MT training program that included didactic sessions, technical skills demonstrations, hands-on practice with virtual simulators (Mentice AB, Gothenburg, Sweden), vascular models with C-arm fluoroscopy units (Siemens, Munich, Germany; Philips, Best, the Netherlands), flow models under camera, independent practice stations, complex case reviews, and expert panel discussions. The final curriculum content was mapped to key competencies in MT, emphasizing patient selection, vascular access strategies, complication management, and post-procedural care. For the Vietnam workshop, pre-recorded live cases were incorporated to enhance practical exposure.

Launched by the WSO in 2020, the GSA is a high-impact implementation meeting aimed at advancing stroke care globally through evidence-based interventions. The meeting typically consists of a 3-day program, beginning with sessions with regional Ministers of Health, a public declaration to combat stroke, signing of the declaration of the fight against stroke by the host country’s health minister, local press conferences or media coverage, and workshops focused on quality-of-care implementation, stroke prevention in primary care, and post-stroke rehabilitation. Health ministers from other invited countries in the region are also typically invited, helping to broaden regional awareness and governmental engagement and participation.

### Eligibility and selection

Candidates, including local practicing neurointerventionalists from the host country and neighboring regions, were invited to apply for the workshop through an open call on multiple online platforms. Candidate selection focused on neurointerventional physicians, defined as individuals with proof of prior specialized training in neurointerventional procedures, working in institutions where stroke treatments were available but either underutilized or not routinely offered. Baseline surveys assessed the current stroke treatment landscape and regional capacity to identify suitable candidates for the workshops. Course faculty implemented a rigorous screening process, reviewing applications and verifying documentation to ensure that candidates were credentialed in their home country and formally trained in neurointervention. In cases where formal certification was lacking, a written recommendation from local senior faculty attesting to the applicant’s role as a practicing “grandfathered” neurointerventionalist was accepted. This program was not intended to replace formal neurointerventional training; rather, its purpose was to improve the MT skills of practicing neurointerventionalists. Final selection prioritized candidates positioned to implement MT in their local settings.

### Ethical considerations

Participants volunteered as part of a WSO Taskforce Quality Improvement initiative. The University of Miami Institutional Review Board granted a waiver under protocol 20260848 for this de-identified educational program analysis; informed consent was not required. To remove financial barriers and maximize access, each workshop covered all participation costs, including travel, food, and lodging for each participant.

### Faculty composition and qualifications

Each workshop featured a core faculty team of two neurointerventionalists, two stroke neurologists, and one radiation technologist, all with extensive experience in neurointerventional procedures and stroke management. Regional workshops included neuroradiology faculty. In addition to core faculty, participating faculty per regional workshop were as follows: Colombia (2 neurologists, 2 neurosurgeons, 4 neuroradiologists), India (3 neurologists, 2 neurosurgeons, 4 neuroradiologists), Türkiye (4 neurologists, 2 neurosurgeons, 4 neuroradiologists), and Vietnam (4 neurologists, 1 neurosurgeon, 2 neuroradiologists). Across all workshops, a total of 24 faculty members contributed to instruction and mentorship.

### Training modalities

The workshop employed a blend of instructional strategies to support MT learning and self-reported procedural confidence. Didactic sessions covered foundational topics, while technical skills demonstrations provided hands-on exposure to key procedural steps. Participants engaged in practical exercises using vascular flow models (silicone, polyacrylate, multi-material 3D-printed, Formlabs Clear Resin) designed to simulate ischemic stroke intervention under fluoroscopy (C-arms; Siemens, Munich, Germany; Philips, Best, the Netherlands). Virtual simulators (Mentice AB, Gothenburg, Sweden) offered a range of MT case scenarios with varying complexity. Additional stations enabled independent practice under faculty supervision. Attendees rotated through stations in small groups to ensure individualized feedback and skill acquisition, practicing techniques such as aspiration alone, stent retriever deployment, combination therapies, anatomical navigation, and device preparation.

### Conference agenda

The agenda for the Global Stroke Alliance in each region was tailored by the planning committee, with the primary language adapted to the host country. In some regions, such as Vietnam, the conference was bilingual (English and Vietnamese). The workshop agenda included didactic lectures on patient selection for MT, strategies for vascular access, prevention and management of MT-related complications, and post-MT intensive care unit management. An example of the customized agenda for each region is provided in Supplementary Table 1.

### Learning objectives

The main learning objectives were to advance knowledge and practical skills in acute stroke care, with a focus on mechanical thrombectomy (MT) and endovascular therapy specifically they included:

- Understanding Patient Selection: Learn criteria and guidelines for selecting patients for MT and thrombolysis, including advanced imaging algorithms and decision-making beyond standard guidelines.
- Mastering Medical Management: Gain expertise in the medical management of acute ischemic stroke and intracerebral hemorrhage.
- Exploring New Research Updates: Review and discuss the latest large core MT trials and emerging technologies in endovascular therapy.
- Developing Technical Skills: Receive hands-on training in neurointerventional procedures, including the use of aspiration devices, stent retrievers, balloon catheters, and strategies to avoid dangerous maneuvers.

### Data management procedures

Survey responses and workshop data were collected and managed using secure, password-protected digital platforms. Data were anonymized prior to analysis to protect participant privacy. Only aggregate, de-identified data are reported. Data analysis was performed using Microsoft Excel Version 16.96.1 (2025) and IBM SPSS Statistics v31.0.2, employing descriptive statistics, chi-square and McNemar’s tests as appropriate.

Data will be made available to qualified investigators upon formal request to the WSO. The manuscript adhered to STROBE checklist recommendations for research reporting (5).

## Results

### Demographics

Over the course of 3 years, we conducted four MT workshops, training 112 locally practicing neurointerventional physicians from 24 countries (see Figure 1). Participants’ professional backgrounds included neurosurgery (31%), neuroradiology (28%), and neurology (41%). Figure 1 illustrates the countries represented by the participating physicians, and Table 1 summarizes their subspecialty backgrounds. All participants provided documentation of formal neurointerventional fellowship training or, in selected cases, a written recommendation confirming their role as a practicing “grandfathered” neurointerventionalist prior to course acceptance.

**Figure 1:**
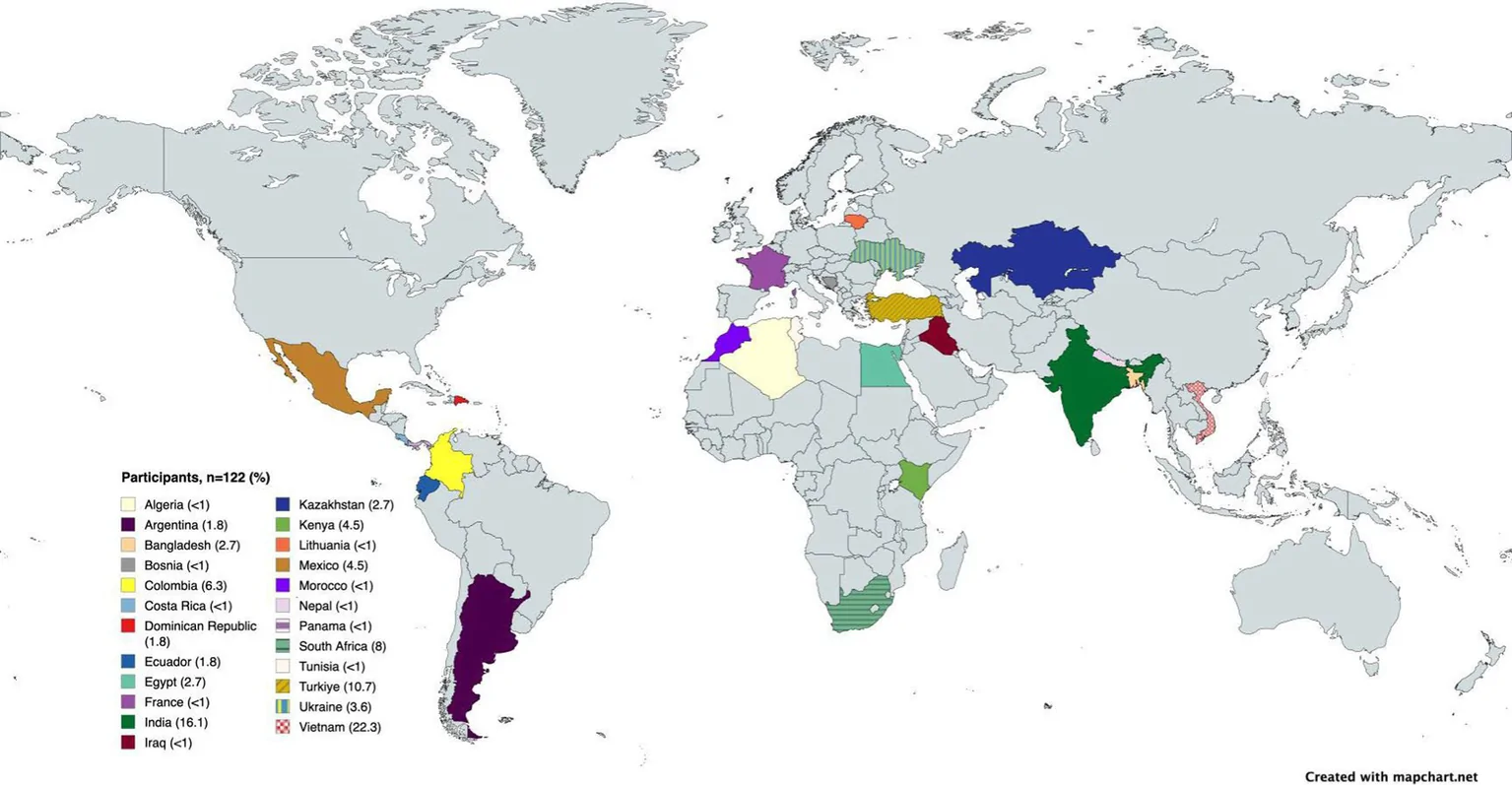
Workshop participant map. Countries represented by participating physicians across the WSO/GSA mechanical thrombectomy workshops.

**Table 1:** Physician background by profession.

Cohort specialties (all regions)	Percentage	Count
Interventional radiology	28%	31
Interventional neurology	41%	46
Interventional neurosurgery	31%	35
Total	100%	112

### Overall self-reported confidence measures

Survey response rates were 81% for the pre-test, 72% for the post-test, and 29% for the three-month follow-up. At the time of submission, 90-day follow-up data for the most recent workshop cohort was not yet available.

In the pre-workshop evaluation, participants reported limited confidence (< 30% “very comfortable”) in 15 of the 16 key MT domains, including patient selection, access systems, anatomical identification and navigation, complication avoidance, and post- procedural ICU management. The only domain rated more highly was intracranial cerebrovascular anatomy, with 31% of participants being “very comfortable” (see Table 2). Immediate post-workshop evaluation demonstrated statistically significant increases in self-reported comfort across all domains (Table 2). Overall, the proportion of participants rating themselves as “very comfortable” increased from 15% pre-workshop to 41% post-workshop.

**Table 2:** Pre-test and post-test summary, independent group.

How comfortable do you feel about	Pre-test (*n* = 91)	Post-test (*n* = 81)	Difference	*p*-value
Selecting the right patient for mechanical thrombectomy?	15%	48%	33%	<0.001
Interpreting advanced imaging of large vessel occlusive stroke?	24%	46%	21%	0.003
Identifying cerebrovascular anatomy?	31%	49%	19%	0.013
Identifying the location of the thrombus?	16%	49%	33%	<0.001
Deciding on the technique for treatment?	9%	41%	32%	<0.001
Knowing the access system?	15%	46%	31%	<0.001
Running the procedure?	9%	41%	32%	<0.001
Navigating the aortic arch?	27%	49%	22%	0.003
Navigating cervical anatomy?	25%	46%	21%	0.005
Navigating intracranial anatomy?	21%	43%	22%	0.002
Navigating to/through the thrombus?	11%	42%	31%	<0.001
Identifying and avoiding harmful techniques?	7%	33%	27%	<0.001
Managing complications?	7%	23%	17%	0.002
Identifying the degree of reperfusion?	9%	36%	27%	<0.001
Leading the team during angiography for MT?	19%	38%	20%	0.004
Operative ICU management?	11%	26%	15%	0.011

% very comfortable, *p*-value Chi Square.

### Paired individual self-reported confidence measures

Although surveys were anonymized, participants could provide a unique 6-digit code to link pre- and post-workshop responses, allowing 44 paired analyses. Among these participants, specialties included endovascular neurosurgery (34%), neuroradiology (27%), interventional neurology (32%), stroke neurology (2%), and other (5%) (Figure 2).

**Figure 2:**
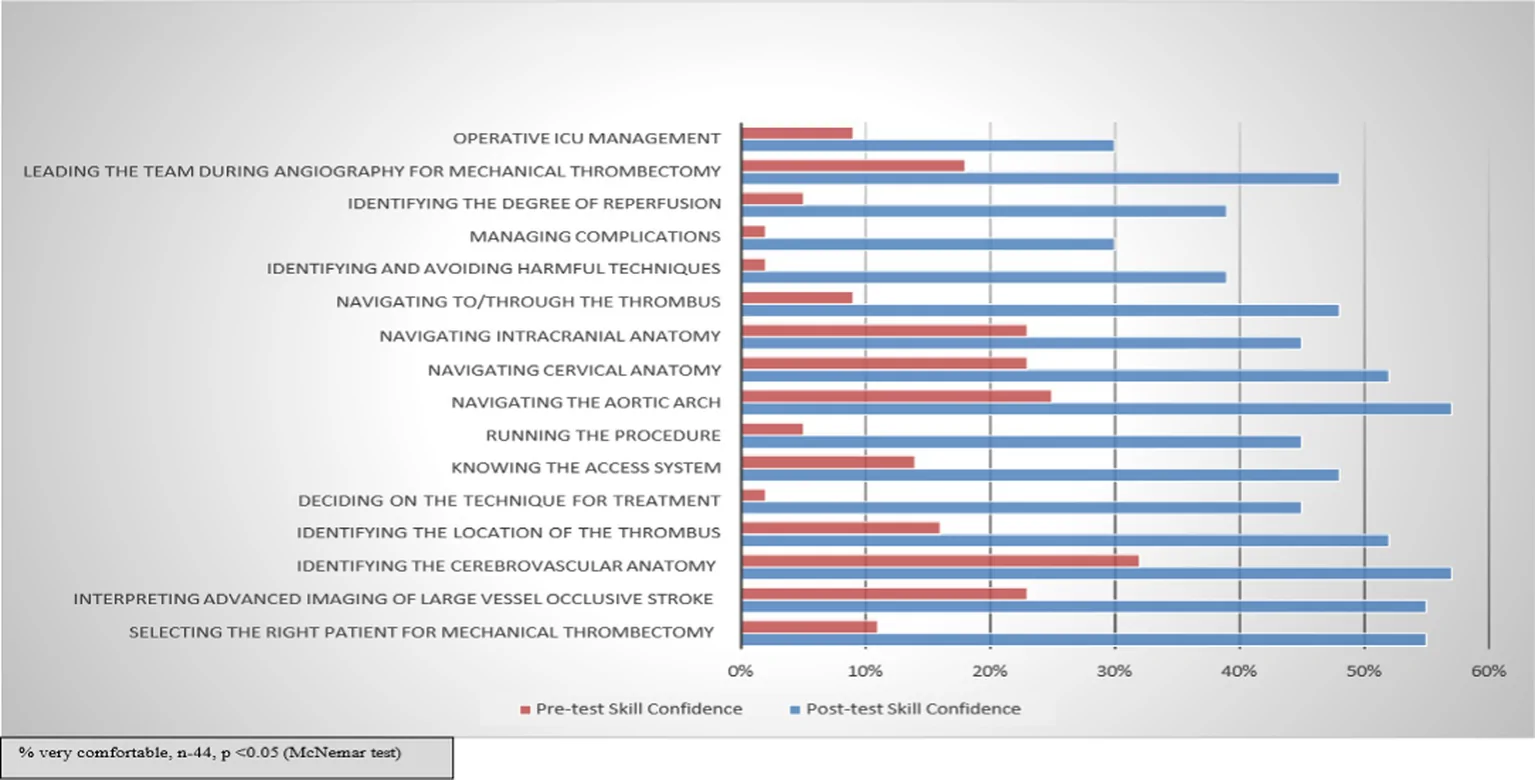
Pre-test and post-test summary, paired group percentage of paired respondents reporting “very comfortable” before and after the workshop across MT confidence domains (*n* = 44; McNemar test, *p* < 0.05). Percentage very comfortable, *n* = 44, *p* < 0.05 (McNemar test).

Confidence increased by an average of 33% across domains, ranging from 23 to 43%.

### Self-reported confidence differences between regions

Analysis of pre- and post-test responses showed improvements in the proportion of participants reporting “Very Comfortable” across all regions. Comparisons between India,

Latin America, and Türkiye revealed no statistically significant differences (Chi-square test, *p* > 0.05 for all datasets). Given the small subgroup sizes, these findings should be interpreted as absence of statistically significant regional differences rather than evidence of equivalent magnitude of change across regions.

### Self-reported confidence differences between specialties

Statistical comparisons for the outcomes between specialties were not conducted. The number of participants in each specialty group was too small to yield reliable or meaningful results. Small sample sizes in these subgroups would limit the validity of statistical testing.

### Three-month MT experience follow-up

Following training, 32 participants (28.6% response rate) completed a follow-up survey to assess changes in practice and identify barriers to MT. During this period, 37.5% of physicians reported an increase in the total number of thrombectomy procedures performed while 13% who had not performed any MTs in the prior 12 months had successfully performed MTs post workshop. Significant changes in procedural technique were also observed. Prior to training, aspiration alone was the most frequently used (25%), followed by combined stent retriever and aspiration (16%), varied techniques (22%), stent retrievers alone (16%), and suction only (3%). After training, practice patterns shifted substantially: 75% of physicians preferred the combined stent retriever and aspiration approach, 13% used aspiration alone, 3% employed varied techniques, 3% reported no specific preference, and 3% reported “other.”

### Barriers

The most frequently reported barriers to performing MT included limited public awareness (66%), procedural costs (56%), hospital administration constraints (25%), limited reimbursements (25%), risk of complications (13%), insufficient staff (13%), lack of neurointerventionalist coverage (13%), and inadequate procedural knowledge (9%).

These findings indicate that lack of procedural knowledge represented only one component of the implementation gap, whereas financial, administrative, staffing, and public awareness barriers were more commonly reported and may limit the impact of education alone.

## Discussion

This study demonstrates that simulation-based MT workshops can be implemented across diverse low- and middle-income or resource-limited regions as part of a broader WSO/GSA capacity-building framework. The workshops were associated with improvements in self-reported procedural confidence across MT domains, supporting the feasibility and educational value of this model. Our findings align with the growing body of literature supporting high-fidelity simulation (6), At the same time, integration with local advocacy efforts through the WSO and GSA may help address public awareness, policy engagement, reimbursement, and stakeholder coordination—barriers that education alone cannot resolve (7).

The baseline barrier analysis also emphasizes that educational programs should be embedded within broader implementation strategies that include public awareness campaigns, payer and reimbursement advocacy, hospital leadership engagement, workforce planning, and mechanisms for longitudinal procedural tracking.

Because the regional comparison was underpowered, the lack of statistically significant differences should not be interpreted as evidence of equivalence across regions.

Importantly, the observation that some centers reported little or no increase in MT volume after training should not be interpreted solely as a negative finding; rather, it helps identify settings where technical education is necessary but insufficient without parallel investments in public education, referral pathways, reimbursement, staffing, and institutional support.

## Strengths

A major strength of this analysis is its inclusion of a large and geographically diverse cohort of participating neurointerventional physicians from 24 countries from primarily low- and middle-income or resource-limited regions worldwide, a scale not previously reported to our knowledge worldwide. Other similar studies had up to eight countries and a fewer total number of physicians (8). Additional workshops have been described in the literature; however these do not include MT simulation and were limited to a single geographic region (9).

More recent studies have explored the role of virtual reality for MT training (10). These efforts have primarily been conducted at single centers in high-income countries with trainees traveling from other high-income countries to the single teaching site. While valuable, such models may have limitations in addressing the substantial global disparities in access to MT, particularly in LMICs.

Another strength of this approach is the delivery of training directly within the region. Available evidence suggests that locally based training can contribute to the development and strengthening of stroke systems of care (11). The unique collaboration with the GSA also enables the inclusion of advocacy efforts that may strengthen local teams and infrastructure.

These activities have the potential to increase reimbursement for MT and raise public awareness about the need for acute stroke intervention and the availability of acute stroke treatments within local communities. Importantly, this study provides evidence that a multi-region, simulation-based MT workshop model can be delivered in collaboration with regional partners and is associated with improved self-reported confidence among participating physicians. The observed changes should be interpreted in the context of self-reported survey outcomes rather than objective assessments of procedural competence, procedural quality, or patient-level outcomes. These findings do not independently verify expanded MT access or improved patient outcomes, but they support the educational feasibility of pairing technical training with broader WSO/GSA advocacy and implementation activities.

## Limitations

As with all studies there may be limitations. One major limitation of this study is the low post-workshop follow-up rate, with only 29% of participants responding at 90 days, and the reliance on self-reported data, which may be subject to reporting bias and cannot be independently verified. Additionally, the absence of a centralized thrombectomy registry precludes objective measurement of changes in procedural volumes and patient outcomes. Although the workshops were intended to strengthen stroke treatment infrastructure, this study primarily measured educational outcomes and self-reported changes in clinical practice rather than direct system-level infrastructure endpoints. Therefore, infrastructure improvement should be interpreted as an anticipated effect of the combined WSO/GSA educational, advocacy, and stakeholder-engagement activities rather than as an outcome independently measured by the workshop evaluation alone.

Additionally, while the workshop focuses on MT training, they cannot on their own address broader barriers as it regards to access for thrombectomy. This highlights the need for multifaceted interventions that combine training with advocacy, policy support, and health system improvements to expand access to care.

## Future directions

Expanding access to MT in LMICs will require a central role for advocacy, including raising public awareness, influencing policy, and supporting reimbursement for procedures. To further advance MT education, complementary strategies such as the development of regional thrombectomy registries can enable longitudinal tracking of procedural outcomes and practice changes. Hands-on simulation workshops, in-person and virtual proctorship, and support from regional colleagues and industry partners can help build and sustain procedural proficiency. Advances in technology make it increasingly feasible to implement and maintain these educational programs at lower costs. Collaborative research and strategic partnerships among healthcare providers, governments, medical societies, educational institutions, and industry partners will be essential to evaluate long-term impact, optimize stroke care delivery, and establish sustainable MT programs in resource-limited settings.

## Conclusion

In conclusion, this study demonstrates that simulation-based MT workshops can be implemented across diverse regions and are associated with improved self-reported procedural confidence among participating neurointerventional physicians.

The integration of these workshops with WSO/GSA advocacy activities may support broader capacity-building by engaging regional stakeholders and increasing awareness, but the present analysis does not independently verify changes in MT access, procedural quality, or patient outcomes.

Accordingly, the findings should be interpreted as evidence of educational feasibility and improved self-reported confidence rather than definitive proof of expanded access to MT.

Future work should incorporate verified procedural volumes, reperfusion metrics, patient-level outcomes, and regional thrombectomy registries to determine whether training-linked capacity-building efforts translate into measurable improvements in MT access and stroke care delivery.

## Data Availability

The raw data supporting the conclusions of this article will be made available by the authors to qualified investigators upon reasonable request, without undue reservation.
