# Supplementary material for: World Stroke Organization mechanical thrombectomy workshops combined with the Global Stroke Alliance builds regional capacity
Source: Front Neurol. 2026 Oct 2;17:1866027. doi: 10.3389/fneur.2026.1866027 (PMC13672226; doi:10.3389/fneur.2026.1866027)
Supplement: Supplementary file 1 [file Supplementary_File_1.pdf]

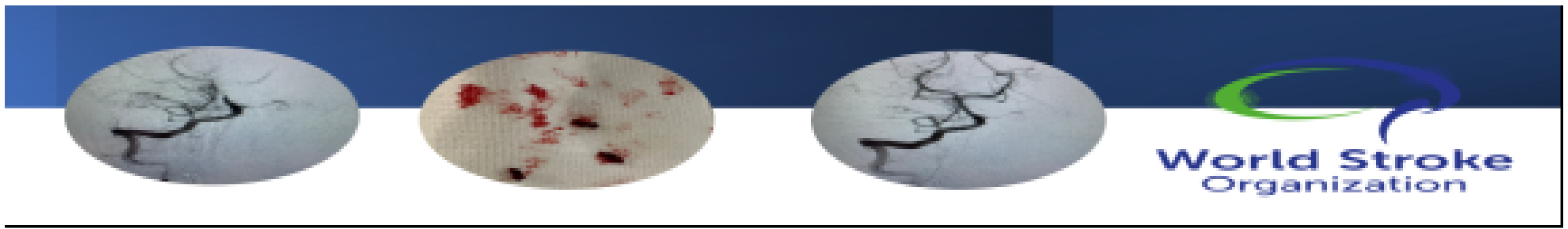

World Stroke Organization
Mechanical Thrombectomy Skills Workshop
In conjunction with the GSA, 2025

Course Directors: Dr. Violiza Inoa, Dr. Ryna Then, Dr. Nitin Goyal and Gillian Gordon Perue
on behalf of the World Stroke Organization Implementation Taskforce.

Dates: July 24-26, 2025
Locations: Hotel Intercontinental Landmark 72, Hanoi Vietnam

Introduction
Worldwide, acute ischemic stroke is the second leading cause of death and the leading cause of adult-onset disability. Mechanical thrombectomy is the most powerful tool in our treatment arsenal against large vessel occlusive stroke with proven morbidity and mortality benefits. Yet, many patients who need this life saving therapy are unable to access it because of a limited stroke system of care in their region.
Join us for an immersive experience at the forefront of acute stroke care. The World Stroke Organization's Mechanical Thrombectomy Skills Workshop is dedicated to advancing knowledge about acute stroke and endovascular therapy, bringing experts in the field of stroke and Neurointervention from diverse backgrounds who will share insights, discuss the latest science, and foster collaboration with you.

Curriculum:
Experience a diverse and engaging learning format which includes didactic lectures, hands on workshops and complex case presentations. Learners will engage with leading experts and peers to enhance their knowledge of both endovascular therapy and stroke care in your region.

1. Over 22.5-hour intensive curriculum which combines both didactic and physician skills sessions.
2. Press Conference to raise public awareness and public education about stroke.
3. Neurointerventional hands on workshops: Demonstration, training, and feedback with aspiration devices, Stent retrievers, balloon catheters and avoiding dangerous maneuvers.
4. Didactic Sessions will focus on:
    a. Patient selection for Mechanical Thrombectomy (MT)
    a. Medical management of acute ischemic stroke
    a. Medical management of intracerebral hemorrhages
    a. New large core Mechanical thrombectomy Trials
    a. Tips for successful Endovascular Access

   a. Management of Complications of MT
   a. ICU care of the Pre- and Post- MT
2. Networking Opportunities
   a. Collaborate with professionals from various disciplines from around the world.
   a. Connect with leading experts in the field.
   a. Explore the latest products in the endovascular field.

Intended Audience: This course is designed for practicing Neuroendovascular physicians.

Funding: The first applicants who meet the following criteria will be invited to participate in this prestigious course which is supported by an unrestricted educational grant. Course space is limited.

WSO Global Stroke Alliance and Angels ASKAN Joint Meeting

24th – 26th July 2025, Thursday - Saturday
0830 Hrs to 1700 Hrs (Vietnam)

Hotel Intercontinental Landmark 72

DAY 1: 24TH JULY (THURSDAY)

Date	DAY 1: GSA and Afternoon Workshop Launch<br>Thursday July 24th, 2025
08:30– 11:00 (ICT)	ALL Attendees follow WSO Global Stroke Alliance and angels ASKAN Joint Meeting Agenda.

TIME	TOPIC	SPEAKER
	**Opening Ceremony**	**(Moderator: Dr Ivy Sebastian)**
0830 - 0845	Registration	ALL
0845-0855	Flag raising ceremony	National Anthem
0855 - 0900	Welcome address	General Manager, Boehringer Ingelheim Vietnam
0900 - 0910	Opening speech	Prof. Jeyaraj Pandian, WSO President
0910 – 0920	Address by the Honorable Health Minister, Government of Vietnam with WSO Stroke Center Certification + Angels Regions Award (HCM + Hanoi) and Ambassador of United States for Vietnam	HE Mr Le Thanh Long, Deputy Prime Minister of Vietnam<br>Honorable Health Minister, Mr Dao Hong Lao, Government of Vietnam; Prof Tran Van Vice Health Minister<br>Mr. Marc E. Knapper
0920- 0925	Address by Director of Bach Mai Hospital; President Hanoi Stroke Society; President, Vietnam Stroke Association	Dr Dao Xuan Co, Director, Bach Mai Hospital; Dr Mai Ton, President of VN Stroke Association; Dr President, Vietnam Stroke Association
0925- 0935	Address by Director, Division of Programs for Diseases Control, WHO Western Pacific Regional Office	Dr Huong Thi Giang Tran, Director, Division of Programs for Diseases Control, WHO Western Pacific Regional Office
0935 – 0945	Signing of Global Declaration on Stroke	WSO and Deputy Prime Minister of Vietnam
0945 – 1000	Group Photo	All

Session 1: Advocacy meeting with Health Ministers and their representatives and delegates (Moderator: Deidre de Silva)		
1010 - 1025	Introduction by WSO President<br><br>Making Stroke a National Priority (Problem: Burden of stroke in Western Pacific region)	Prof Jeyaraj Pandian, WSO President
1025 - 1040	Global Stroke Action Coalition: the new advocacy initiative	WSO
1040 – 1050	Generating insights for Improvement: Evaluating Ischemic stroke care in Singapore through a value-driven care approach	Dr Albert Ty: Singapore Ministry of Health
1050 – 1100	Angels Initiative –Improving access to healthcare for stroke patients	Sylvia Kang, Head of Sustainability Development (ASKAN), Boehringer Ingelheim

11:00-11:15 (ICT)	**NIR Workshop Tea Break**
	**Neurointerventional Symposium and Hands on Workshop in Collaboration with Penumbra**<br>**Ballroom 1**<br><br>**Faculty:** Dr. Violiza Inoa, Nicole Cancelliere, Dr. Gillian Gordon Perue, Dr. Nitin Goyal, Dr. Laetitia de Villiers, Dr. Ryna Then, Dr. Ruchi Kabra, Dr Rajsrinivas Parthasarathy, and Prof Vu Dang Luu.<br><br>**Location:**<br>**11:15 - 11:25 Welcome and Introduction**<br>Dr. Violiza Inoa, USA<br>**11:30 – 11:50 Impact of Mechanical Thrombectomy Training Workshops**<br>Dr. Violiza Inoa, USA<br>**11:50- 12:05: Course Orientation and Completion of initial survey**<br>Dr. Gillian Gordon Perue, USA<br><br>**12:05 – 12:35: Patient Selection for Mechanical Thrombectomy and Thrombolysis: the guidelines and beyond**<br>Dr. Ryna Then, USA<br><br>**12:35 – 13:00: Imaging Algorithms: Variables and Alternatives**<br>Dr. Gillian Gordon Perue, USA
13:00 – 14:00 (ICT)	**LUNCH** (in the room)<br>Peer Interactions and Networking session

14:00- 17:30 (ICT)<br>NIR Workshop Opening	**Neurointerventional Symposium and Hands on Workshop**<br><br>Welcome to NIR Sessions and Intro of Rotations<br>Nicole Cancelliere, Canada<br><br>**Location:** Ballroom 1
	**14:00 – 15:30: NIR Hands on Session with Camera Models**(1.5 hours)<br><br>**Faculty:** Dr. Violiza Inoa, Nicole Cancelliere, Dr. Gillian Gordon Perue, Dr. Nitin Goyal, Dr. Laetitia de Villiers, Dr. Ryna Then, Dr. Ruchi Kabra, Dr Rajsrinivas Parthasarathy,and Prof Vu Dang Luu.
	**15: 30- 16:00 TEA BREAK**<br><br>---<br><br>**16:00 – 17:30: NIR Hands on Session with Camera Models**<br><br>**Faculty:** Dr. Violiza Inoa, Nicole Cancelliere, Dr. Gillian Gordon Perue, Dr. Nitin Goyal, Dr. Laetitia de Villiers, Dr. Ryna Then, Dr. Ruchi Kabra, Dr Rajsrinivas Parthasarathy,and Prof Vu Dang Luu.
17:30- 19:00 (ICT)	Angels ASKAN Award session
Date	DAY 2: In Dept Workshops<br>Friday July 25th, 2025
07:00 – 07:30 (ICT)	Breakfast
**7:30 - 8:00** (ICT)	**Bus leaves hotel at 07:30 AM**<br><br>**Day 2 Travel to Hospital Bach Mai, Vietnam**<br>Day 2 Hospital Bach Mai<br><br>**Neurointerventional Symposium and Hands on Workshop**
8:30 – 12:00 (ICT)	**08:30 – 08:35 Welcome to Day 2**<br>Dr. Gillian Gordon Perue, USA

	**08:35 – 08:50 Tips for endovascular access**<br>Dr. Violiza Inoa, USA<br><br>**08:50 – 09:10 Hints for tailoring thrombectomy: What's the Best Strategy**<br>Dr. Ruchi Kabra, Australia<br><br>**09:10 – 09:30 Challenging Thrombectomy**<br>Dr. Nitin Goyal, USA<br><br>**09:30-09:50 Challenging Case presentation**<br>Dr Rajsrinivas Parthasarathy<br>QA Panel Discussion: Moderated by course faculty<br><br>**09:50 -11:00 NIR MT Course: IR Workshop: Participants hands MT Skills Stations on rotations Faculty:** Dr. Violiza Inoa, Nicole Cancelliere, Dr. Nitin Goyal, Dr. Laetitia de Villiers, Dr. Ruchi Kabra, Dr Rajsrinivas Parthasarathy, and Prof Vu Dang Luu.<br><br>**11:00- 11:15 TEA BREAK**<br><br>**11:30- 13:00 NIR MT Course: IR Workshop: Participants hands MT Skills Stations on rotations** Dr. Violiza Inoa, Nicole Cancelliere, Dr. Nitin Goyal, Dr. Laetitia de Villiers, Dr. Ruchi Kabra, Dr Rajsrinivas Parthasarathy, and Prof Vu Dang Luu.
13:00 – 14:00 (ICT)	**Lunch**
14:00 – 15:30PM (ICT)	**NIR MT Course: IR Workshop: Participants hands MT Skills Stations on rotations**<br><br>Dr. Violiza Inoa, Nicole Cancelliere, Dr. Nitin Goyal, Dr. Laetitia de Villiers, Dr. Ruchi Kabra, Dr Rajsrinivas Parthasarathy,and Prof Vu Dang Luu.
15:30- 16:00 (ICT)	**TEA Break**
16:00- 17:00 (ICT)	**NIR MT Course: IR Workshop: Participants hands MT Skills Stations on rotations**<br>Dr. Violiza Inoa, Nicole Cancelliere, Dr. Nitin Goyal, Dr. Laetitia de Villiers, Dr. Ruchi Kabra,Dr Rajsrinivas Parthasarathy, and Prof Vu Dang Luu.

17:00 (ICT)	Bus back to the hotel
Date	DAY 3: Complication Management and Challenging MT<br>Saturday July 26th, 2025
07:00 – 8:00 AM (ICT)	Breakfast
08:00 – 08:30 (ICT)	**Bus leaves Hotel at 08:00 am**<br>**Day 3 Travel to Hospital Bach Mai, Vietnam**
08:30-08:35 am (ICT)	Welcome to Day 3 Hospital Bach Mai<br>**Neurointerventional Symposium and Hands on Workshop**<br>Ryna Then, USA
08:35 – 10:00 (ICT)Mechanical Thrombectomy Workshop Didactics	**08:35 – 09:05 Live Case Demonstration Dr. Vitor Pereira, Canada** (Friday 9:35 PM Canada Time)<br><br>**09:05 - 09: 35 MT Complications - Prevention and Management**<br>Dr. Laetitia de Villiers, Australia<br><br>**09:35- 10:00 Post Thrombectomy and ICU Care**<br>Nitin Goyal, USA<br><br>**10:20 - 10:40 Round Table Discussion: Complex Mechanical Thrombectomy Local Cases** Prof Vu Dang Luu,<br><br>Panelists: Dr. Nitin Goyal and Dr. Violiza Inoa
10:00 – 11:00 (ICT)	**NIR MT Course: IR Workshop: Participants hands MT Skills Stations on rotations**
11:00 – 11:15	**TEA Break**
11:15-13:00 (ICT)	Conference Award Ceremony, (attendees awarded with Certificates)<br><br>***Photos with Faculty***<br><br>**End of Conference Survey,** Gillian Gordon Perue, USA<br><br>**Closing Remarks**

	Dr. Violiza Inoa, USA
