# Supplementary material for: World Stroke Organization mechanical thrombectomy workshops combined with the Global Stroke Alliance builds regional capacity
Source: Front Neurol. 2026 Oct 2;17:1866027. doi: 10.3389/fneur.2026.1866027 (PMC13672226; doi:10.3389/fneur.2026.1866027)
Supplement: Supplementary file 3 [file Supplementary_file_3.pdf]

2025 World Stroke Organization Mechanical Thrombectomy Workshops.

Annually, over 17 million people worldwide suffer from acute ischemic stroke. Of these, approximately 4 to 6 million could potentially benefit from Mechanical Thrombectomy (MT), yet only about 140,000 undergo this treatment, with nearly 70% of these procedures occurring in the US and Western Europe.

Mortality and disability from stroke are notably higher in low- to middle-income countries, where there is a marked disparity in access to MT. This suggests a significant number of patients globally who are underserved by MT treatment.

We invite you to an immersive experience at the cutting edge of acute stroke care. The WSO Mechanical Thrombectomy Workshop, in partnership with the Global Stroke Alliance, aims to expand knowledge on acute stroke and endovascular therapy. It brings together experts from various backgrounds in stroke and endovascular diseases to share insights, discuss recent scientific developments, and encourage collaboration.

Curriculum:

The program offers a dynamic and interactive learning format that includes didactic lectures, hands-on workshops, and complex case studies. Participants will interact with leading experts and peers to deepen their understanding of endovascular therapy and the advancement of care systems in their regions.

1. An intensive 22.5-hour curriculum that integrates didactic learning with practical physician skills sessions.

2. This MT workshop will be conducted in collaboration with the Global Stroke Alliance meeting to enhance public awareness and education about stroke.

3. Tailored to individual needs during small group sessions led by expert faculty, featuring Neurointerventional hands-on workshops.

* Indicates required question

1. Email *

____

2. Name *

3. Title/Designation

4. Hospital Affiliation(s)
(You may write more than one hospital, if applicable separated by commas)

5. City

6. Country

7. Email Address *

8. Telephone

9. What is your Specialty? *

Mark only one oval.

- Interventional Neurologist
- Endovascular Neurosurgeon
- Interventional Neuroradiologist
- Other Interventionalist
- Stroke Neurologist
- Other Stroke Care Provider
- Stroke Medical Director

10. Did you complete formal neuroendovascular training? *

Mark only one oval.

- Yes
- No

11. How many years of neuroendovascular experience do you have after training? *

Mark only one oval.

- <1 year
- 1-2 years
- 3-5 years
- >5 years

12. Please select your hospital type *

Mark only one oval.

- ( ) Academic Public/Government Institution
- ( ) Academic Private Institution
- ( ) Non-Academic Public/Government Institution
- ( ) Non-Academic Private Institution
- ( ) Others

13. How many stroke patients are admitted to your institution (average number) every year? *

Mark only one oval.

- ( ) None
- ( ) 1-50
- ( ) 51-100
- ( ) >100

14. Please select the acute ischemic stroke treatments offered at your institution: *

Mark only one oval.

- ( ) None
- ( ) IV thrombolysis.
- ( ) Mechanical thrombectomy.

15. If Intravenous thrombolysis, how many cases per year? *

Mark only one oval.

- < 10
- 10-50
- 50-100
- >100

16. If Mechanical Thrombectomy, how many cases per year? *

Mark only one oval.

- < 10
- 10-50
- 50-100
- >100

17. How many Mechanical thrombectomy procedures have you personally performed over the past year? *

Mark only one oval.

- < 10
- 10-50
- 50-100
- >100

18. Who does your scrubbed team consist of? You can select all that apply: *

Check all that apply.

- [ ] I scrub alone
- [ ] Nurses
- [ ] Techicians
- [ ] Fellow
- [ ] Students

19. How many team members are in your scrubbed team? *

Mark only one oval.

- ( ) I scrub alone
- ( ) 1-3
- ( ) >4

20. Would your scrub team be interested in joining this course?

Mark only one oval.

- ( ) Yes
- ( ) No
- ( ) Maybe

21. If yes, provide the name and contact information of your neurointerventional nurse or technician below:

____

22. Please select all the resources currently available for stroke care at your institution. Select all that apply. *

Check all that apply.

- [ ] Computerized Tomography
- [ ] Computerized Tomography Angiography
- [ ] Computerized Tomography Perfusion
- [ ] Magnetic Resonance Imaging
- [ ] Magnetic Resonance Angiography
- [ ] Magnetic Resonance Perfusion

23. Is your hospital certified as a stroke center? *

Mark only one oval.

- ( ) Yes
- ( ) No
- ( ) Not applicable

24. Does your hospital have a Stroke Medical Director? *

Mark only one oval.

- ( ) Yes
- ( ) No
- ( ) Not applicable

25. Does your hospital have a Stroke Coordinator? *

Mark only one oval.

- Yes
- No
- Not applicable

26. If your hospital is a certified stroke center, what is the level of certification?

Mark only one oval.

- Primary
- Advanced
- Other
- Not applicable

27. If your hospital is stroke Certified, what is the name of the certifying body?

____

28. How is your hospital classified? *

Mark only one oval.

- Primary Hospital or Nursing Home
- Secondary Hospital
- Single super specialty hospitals and other specialties are provided as an allied service.
- Multi super specialty hospital including ICU and rehab.
- Tertiary referral center

29. Do you think a mechanical thrombectomy training course (with didactics and hands on sessions) would be valuable for Neurointerventional physicians? *

Mark only one oval.

- Yes
- No
- Maybe

30. Would you be interested in attending such a course? *

Mark only one oval.

- Yes
- No
- Maybe

31. Please select the following subjects you would be interested in discussing as part of a mechanical thrombectomy training course (didactic sessions): *

Check all that apply.

- [ ] General stroke diagnosis
- [ ] Stroke triage
- [ ] Advanced imaging for stroke treatment selection
- [ ] Status of stroke care in India
- [ ] Status of mechanical thrombectomy
- [ ] Technical approaches to mechanical thrombectomy
- [ ] Complications during mechanical thrombectomy
- [ ] Patient care post mechanical thrombectomy
- [ ] Challenging mechanical thrombectomy cases
- [ ] New mechanical thrombectomy studies
- [ ] Stroke Health Medical Economics
- [ ] Building Stroke Care Networks
- [ ] Telemedicine in Acute Stroke

32. Which technical aspects of MT are you most interested in learning about? Select all that apply: *

Check all that apply.

- [ ] Access/difficult access
- [ ] Catheter preparation (i.e. balloon guide catheter)
- [ ] Stent retriever technique
- [ ] Aspiration technique
- [ ] Combination therapy
- [ ] Other: ______________________

33. What are the main limiting factors to perform MT in your center? Select all that apply: *

Check all that apply.

- [ ] Late windows (patients don't arrive on time)
- [ ] No triaging protocols for large vessel occlusion
- [ ] Lack of advanced imaging (CTA, CTP, MR, MRP)
- [ ] Lack of ancillary staff to support the cases (RNs, Techs)
- [ ] Lack of appropriate devices to perform the MT
- [ ] Lack of neuroendovascular physician support
- [ ] Lack of reimbursement for the MT
- [ ] Upfront costs of MT procedures to the patient
- [ ] Delayed on medical insurance approval
- [ ] No adequate post MT care
- [ ] Limited administrative support from your institution

34. *On a scale of 1-5, how comfortable do you currently feel about:* * Selecting the right patient for Mechanical Thrombectomy?

Dropdown

Mark only one oval.

- Very Uncomfortable (1)
- Uncomfortable (2)
- Neutral
- Comfortable (4)
- Very Comfortable

35. *On a scale of 1-5, how comfortable do you currently feel about:* Interpreting Advanced Imaging of Large Vessel Occlusive Stroke? * Dropdown

Mark only one oval.

- Very Uncomfortable (1)
- Uncomfortable (2)
- Neutral
- Comfortable (4)
- Very Comfortable

36. *On a scale of 1-5, how comfortable do you currently feel about:* Identifying the Cerebrovascular anatomy? * Dropdown

Mark only one oval.

- Very Uncomfortable (1)
- Uncomfortable (2)
- Neutral
- Comfortable (4)
- Very Comfortable

37. *On a scale of 1-5, how comfortable do you currently feel about:* Identifying the location of the thrombus? * Dropdown

Mark only one oval.

- Very Uncomfortable (1)
- Uncomfortable (2)
- Neutral
- Comfortable (4)
- Very Comfortable

38. *On a scale of 1-5, how comfortable do you currently feel about:* Deciding on the technique for treatment? * Dropdown

Mark only one oval.

- Very Uncomfortable (1)
- Uncomfortable (2)
- Neutral
- Comfortable (4)
- Very Comfortable

39. *On a scale of 1-5, how comfortable do you currently feel about:* Knowing the access system? * Dropdown

Mark only one oval.

- Very Uncomfortable (1)
- Uncomfortable (2)
- Neutral
- Comfortable (4)
- Very Comfortable

40. *On a scale of 1-5, how comfortable do you currently feel about:* Running the procedure? * Dropdown

Mark only one oval.

- Very Uncomfortable (1)
- Uncomfortable (2)
- Neutral
- Comfortable (4)
- Very Comfortable

41. *On a scale of 1-5, how comfortable do you currently feel about:* Navigating the aortic arch? * Dropdown

Mark only one oval.

- Very Uncomfortable (1)
- Uncomfortable (2)
- Neutral
- Comfortable (4)
- Very Comfortable

42. *On a scale of 1-5, how comfortable do you currently feel about:* Navigating cervical anatomy? * Dropdown

Mark only one oval.

- Very Uncomfortable (1)
- Uncomfortable (2)
- Neutral
- Comfortable (4)
- Very Comfortable

43. *On a scale of 1-5, how comfortable do you currently feel about:* Navigating intracranial anatomy? * Dropdown

Mark only one oval.

- Very Uncomfortable (1)
- Uncomfortable (2)
- Neutral
- Comfortable (4)
- Very Comfortable

44. *On a scale of 1-5, how comfortable do you currently feel about:* Navigating to/through the thrombus? * Dropdown

Mark only one oval.

- Very Uncomfortable (1)
- Uncomfortable (2)
- Neutral
- Comfortable (4)
- Very Comfortable

45. *On a scale of 1-5, how comfortable do you currently feel about:* Identifying and avoiding harmful techniques? * Dropdown

Mark only one oval.

- Very Uncomfortable (1)
- Uncomfortable (2)
- Neutral
- Comfortable (4)
- Very Comfortable

46. *On a scale of 1-5, how comfortable do you currently feel about:* Managing Complications? * Dropdown

Mark only one oval.

- Very Uncomfortable (1)
- Uncomfortable (2)
- Neutral
- Comfortable (4)
- Very Comfortable

47. *On a scale of 1-5, how comfortable do you currently feel about:* Identifying the degree of reperfusion? * Dropdown

Mark only one oval.

- Very Uncomfortable (1)
- Uncomfortable (2)
- Neutral
- Comfortable (4)
- Very Comfortable

48. *On a scale of 1-5, how comfortable do you currently feel about:* Leading the team during angiography for Mechanical Thrombectomy? * Dropdown

Mark only one oval.

- Very Uncomfortable (1)
- Uncomfortable (2)
- Neutral
- Comfortable (4)
- Very Comfortable

49. *On a scale of 1-5, how comfortable do you currently feel about:* Post Operative ICU Management? * Dropdown

Mark only one oval.

- Very Uncomfortable (1)
- Uncomfortable (2)
- Neutral
- Comfortable (4)
- Very Comfortable

This content is neither created nor endorsed by Google.

Google Forms
